# Ferroptosis in tumors and its relationship to other programmed cell death: role of non-coding RNAs

**DOI:** 10.1186/s12967-023-04370-6

**Published:** 2023-07-29

**Authors:** Qi Zhang, Xinfeng Fan, Xinyu Zhang, Shaoqing Ju

**Affiliations:** 1 Medical School of Nantong University, Nantong University, Department of Laboratory Medicine, Affiliated Hospital of Nantong University, Nantong, 226001 Jiangsu China; 2 Research Center of Clinical Medicine, Affiliated Hospital of Nantong University, Nantong, 226001 Jiangsu China; 3 Present Address: Department of Medical School of Nantong University, No.19, Qixiu Road, Nantong, 226001 Jiangsu China; 4 Present Address: Department of Laboratory Medicine, Affiliated Hospital of Nantong University, No.20, Xisi Road, Nantong, 226001 Jiangsu China

**Keywords:** Ferroptosis, Programmed cell death, Non-coding RNA, Tumor, Autophagy

## Abstract

Programmed cell death (PCD) plays an important role in many aspects of individual development, maintenance of body homeostasis and pathological processes. Ferroptosis is a novel form of PCD characterized by the accumulation of iron-dependent lipid peroxides resulting in lethal cell damage. It contributes to tumor progression in an apoptosis-independent manner. In recent years, an increasing number of non-coding RNAs (ncRNAs) have been demonstrated to mediate the biological process of ferroptosis, hence impacting carcinogenesis, progression, drug resistance, and prognosis. However, the clear regulatory mechanism for this phenomenon remains poorly understood. Moreover, ferroptosis does not usually exist independently. Its interaction with PCD, like apoptosis, necroptosis, autophagy, pyroptosis, and cuproptosis, to destroy cells appears to exist. Furthermore, ncRNA seems to be involved. Here, we review the mechanisms by which ferroptosis occurs, dissect its relationship with other forms of death, summarize the key regulatory roles played by ncRNAs, raise relevant questions and predict possible barriers to its application in the clinic, offering new ideas for targeted tumour therapy.

## Introduction

Ferroptosis is a novel model of cell death, as defined in 2012 [1]. It is distinguished from other types of deaths by apoptosis, necroptosis, autophagy, pyroptosis, and cuproptosis [1, 2]. Its main morphological manifestations are shrinking mitochondria, increased membrane density, and fewer cristae. In recent years, research into ferroptosis has expanded tremendously. Numerous scientific breakthroughs have been gained in oncology, and targeting ferroptosis has become a potential cancer therapy.

Although each programmed cell death (PCD) has a unique mechanism of occurrence and cellular and biochemical properties, mixed types of cell death seem more prevalent than single types of death in most cells. Some of their components and factors are synergistic. Exploring how ferroptosis interacts with other PCDs at the molecular level and identifying and integrating shared pathways will open new areas for systematic research [3].

Ninety-eight percent of the human genome is transcribed into RNAs that do not encode proteins, known as non-coding RNAs (ncRNAs) [4]. Evidence suggests they are vital in basic biological processes like growth and development and almost every human disease, particularly cancer [5, 6]. At the same time, ncRNAs have been shown to be involved in the biology of ferroptosis and, in turn, influence tumour progression. This implies that ncRNA-based targeted iron death therapy is a promising novel anti-cancer therapy. However, the mechanisms by which ncRNAs regulate ferroptosis are still poorly understood. Furthermore, the role of ncRNAs in ferroptosis has not been fully defined.

In this review, we provide new ideas for targeting ncRNAs in ferroptosis-related therapeutic strategies by systematically summarizing ferroptosis mechanisms and the progress of ncRNA targeting of ferroptosis signaling pathways in tumors, paying particular attention to the interactions between ferroptosis and other PCDs.

## Mechanism of ferroptosis

**Fig. 1:**
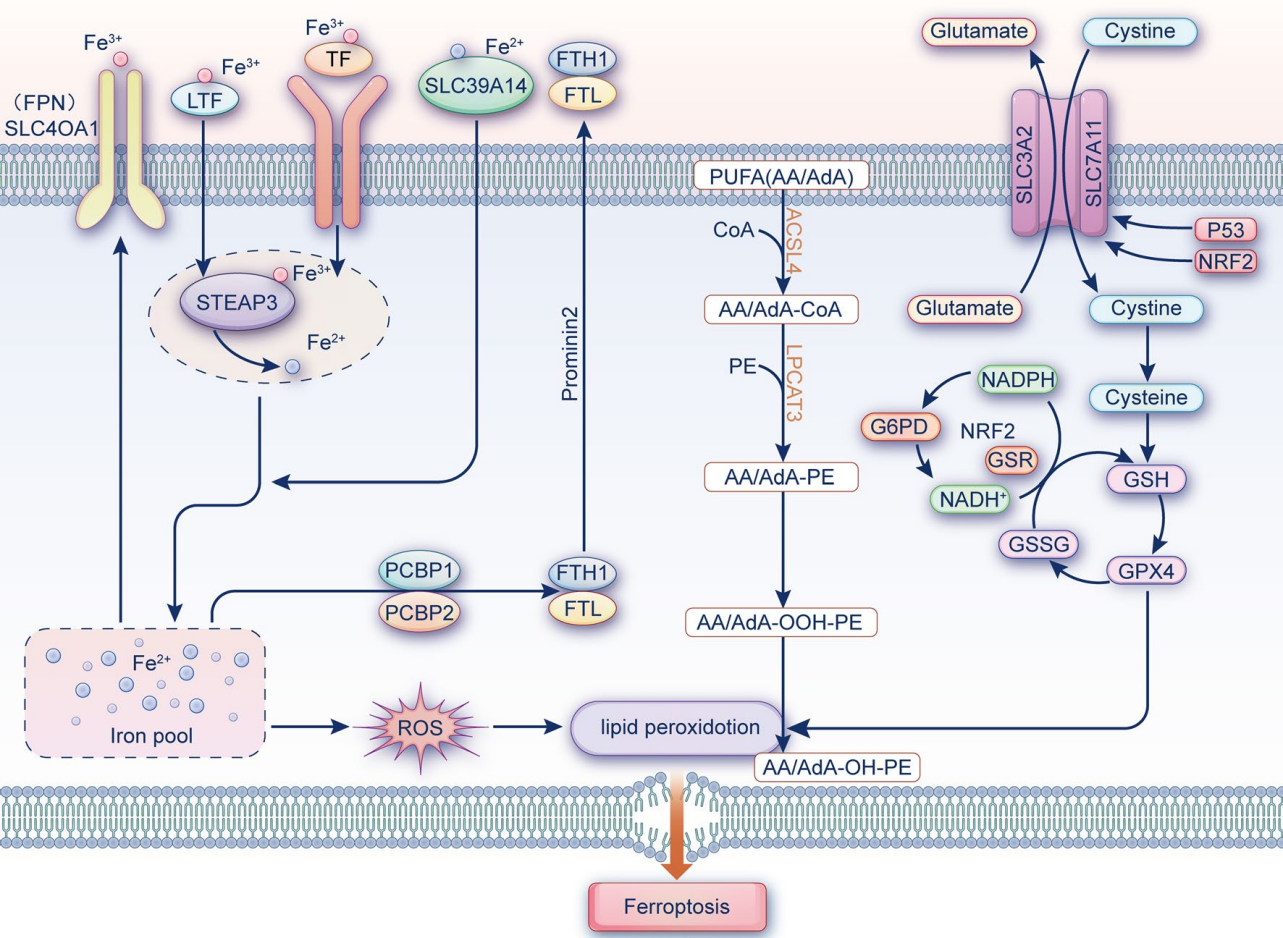
The core molecular mechanisms of ferroptosis. The regulatory pathways of ferroptosis are divided into iron metabolism, lipid metabolism and the system xc-/GSH/GPX4 axis. Iron metabolism: Transferrin (TF); Transferrin receptor 1 (TFRC); ferroportin (FPN); Ferritin heavy chain 1 (FTH1); Ferritin light chain (FTL); solute carrier family 39 member 14 (SLC39A14); Six transmembrane epithelial antigen of protein 3 (STEAP3); Poly (RC) binding protein 1/2 (PCBP1/2); Reactive oxygen species (ROS); Lipid metabolism: Polyunsaturated fatty acid (PUFA); Long chain acyl CoA synthetase 4 (ACSL4); Lysophosphatidylcholine acyltransferase 3 (LPCAT3); Phosphatidylethanolamine (PE); arachidonic acid (AA); adrenic acid (AdA), coenzyme A (CoA); system xc-/GSH/GPX4 axis: Solute carrier family member 7A11 (SLC7A11); Solute carrier family member 3A2 (SLC3A2); Glutathione (GSH); glutathione-disulfide reductase (GSR); glucose 6-phosphate dehydrogenase (G6PD); Glutathione peroxidase 4 (GPX4); oxidized glutathione (GSSG); nicotinamide adenosine dinucleotide hydrogen phosphate (NADPH); Nuclear factor E2 related factor 2 (NRF2)

Ferroptosis is a novel form of cell death regulation that relies on iron ion-mediated oxidative damage. Ferroptosis may be triggered when intracellular iron ion-dependent reactive oxygen species (ROS) accumulate in excess and glutathione peroxidase 4 (GPX4) scavenging is diminished, resulting in an imbalance in the homeostasis of ROS production and degradation, i.e. a redox imbalance between intracellular oxidants and antioxidants [7]. Current molecular mechanisms of ferroptosis include glutathione (GSH) depletion, lipid peroxidation, and impaired iron metabolism (Fig. 1). The various molecules and signals involved in iron metabolism and lipid peroxidation will be discussed below.

### The canonical system XC-/GSH/GPX4 pathway

Amino acid metabolism is an important part of the metabolic cycle of organisms, and abnormal amino acid metabolism is closely related to ferroptosis. Cystine/glutamic acid reverse transporter (system Xc-) plays an important role in maintaining the balance and distribution of amino acids and is a very important antioxidant system in cells. Its inactivation of the cellular antioxidant system by downregulation or inhibition of the Cystine/glutamic acid reverse transporter (system Xc-) is a major determinant of the suceptibility to ferroptosis. System XC- consists of the light chain xCT/solute carrier family 7 member 11 (SLC7A11) and the heavy chain 4F2hc/solute carrier family 3 member 2 (SLC3A2), and SLC3A2 is a chaperone that facilitates momemnt of SLS7A11 to the plasma surface and SLC7A11 forms the transport channel in its oxidated form [8]. Cystine is transported intracellularly by system XC- then transformed into cysteine. Cysteine is the rate-limiting amino acid for GSH (a vital intracellular antioxidant) production. Moreover, GPX4, a member of the selenium family containing GPXs, is a recognized negative regulator of ferroptosis. It is an enzyme for the reduction of toxic peroxides (L-OOH) to non-toxic lipid alcohols (L-OH) [9, 10]. It was shown that GSH is an essential cofactor of GPX4 and can influence the GPX4 function [11]. Therefore, system XC-mediated cysteine can also indirectly affect GPX4 activity. Furthermore, GSH synthesis requires the nicotinamide adenosine dinucleotide hydrogen phosphate (NADPH) cycle to supply ATP.

### Lipid metabolism pathway

Lipids are important regulators of cell death, and the accumulation of lipid peroxides is thought to be an important driver of ferroptosis [12]. Although the exact source of lipid peroxides is unknown, polyunsaturated fatty acids (PUFAs) have been identified as an important source. PUFAs are an important component of cell membranes and they can perform many cellular functions by enhancing cell mobility. However, they contain unstable carbon–carbon double bonds that can generate lipid reactive oxygen species, which can cause ferroptosis when accumulated in excess [13]. Among PUFAs, arachidonic acid (AA) and adrenoic acid (ADA) are the 203 main substrates causing lipid peroxidation during ferroptosis [14]. In contrast, acyl-coenzyme A synthase long-chain family member 4 (ACSL4) and lysophosphatidylcholine acyltransferase 3 (LPCAT3) are required for the biosynthesis and remodeling of AA/AdA derivatives. Both can catalyze the formation of AA/AdA-CoA derivatives and AA/AdA-phosphatidylethanolamine (AA/AdA-PE) from free AA/AdA. AA/AdA-PE then synthesizes lipid peroxides AA/AdA-hydroperoxide-PE (AA/AdA-OOH-PE) through enzymatic and non-enzymatic reactions [15]. Lipid peroxides themselves and their degradation products (malondialdehyde (MDA) and 4-hydroxynonenal (4-HNEs)) produce cytotoxicity and cause cell death [16]. Moreover, the degradation process involves cyclooxygenase-2 (COX2) and nicotinamide adenine dinucleotide phosphate oxidases 2 (NOX2), among others [17].

### Iron metabolism pathway

Iron has a dual role in cell growth. Although iron is a trace element essential for cell proliferation, its excessive accumulation can cause cell damage and increase the risk of diseases such as tumors [7]. Iron ions are also an important component in the accumulation of lipid peroxides and the initiation of iron death. The key to iron metabolism is the regulation of iron pool capacity, which mainly includes iron uptake, storage and export.

1. Iron ions are transferred into the cytosol through multiple pathways. In one respect, transferrin (Tf) and lactotransferrin (LTF) store extracellular iron as Fe^3+^, which is then bound to the transferrin receptor (TfR) and another unknown receptor on the cell membrane, and Fe^3+^ is endocytosed to form endo nucleosomes [18, 19]. In the endosome, the metal reductase six transmembrane epithelial antigen of protein 3 (STEAP3) reduces Fe^3+^ to Fe^2+^. On the contrary, solute carrier family 39-member 14 (SLC39A14/ZIP14) and solute carrier family 39-member 8 (SLC39A8/ZIP8) transfer Fe^2+^ directly into the intracellular compartment by transporting non-transferrin-bound iron (NTBI) to the cell membrane [20].
2. Multiple mechanisms maintain the equilibrium of Fe^2+^ in the cytoplasm. Poly C-binding protein 1/2 (PCBP1/2) oxidizes most Fe^2+^ to Fe^3+^, which is stored in ferritin (composed of light chain (FTL) and heavy chain 1 (FTH1)), which itself can be degraded to increase free iron levels; iron regulatory protein (IRP1/2) promotes the free iron utilization in cells in multiple pathways; and heme oxygenase 1 (HO-1), regulated by the nuclear factor E2-related factor 2 (Nrf2 / NFE2L2) gene, catalyzes the degradation of heme to produce Fe^2+^ [21].
3. Iron efflux protein solute carrier family 40 member 1 (SLC40A1/ferroportin1/FPN) and ferritin transfer out protein Prominin2 can facilitate the export of intracellular ferric ions and ferritin [22]. When the intracellular iron metabolic pathway is abnormal, and an unstable iron pool is formed, Fe^2+^ then generates ROS through the Fenton reaction [1] or participates in the iron-containing lipoxygenase activation [23], triggering lipid peroxidation, leading to cell damage. This process is known as ferroptosis.

In conclusion, iron is crucial to the physiological functioning of cells. A lack of iron can cause cells to malfunction, whereas an abundance of iron can cause oxidative stress on cells and ferroptosis.

### Other metabolic pathways

P53, the “star molecule” of oncology, is a double-edged sword in ferroptosis. P53 is a SLC7A11 transcriptional repressor, which increases cellular sensitivity to ferroptosis through SLC7A11 in a GPX4-dependent or non-dependent pathway [24]. Additionally, P53 negatively regulates ferroptosis by acting on dipeptidyl peptidase 4 (DPP4) or by inducing cell cycle protein-dependent kinase inhibitor 1A (CDKN1A/p21) [25].

The transcription factor Nrf2 is involved in antioxidant responses, and various iron and lipid metabolism factors are among its target genes [26]. Thus, Nrf2 can counteract ferroptosis by regulating intracellular iron ion content [27], GPX4 levels [28], and the NAPDH cycle [29].

The flavin protein apoptosis-inducing factor mitochondrial-associated 2 (AIFM2), subsequently renamed ferroptosis inhibitory protein 1 (FSP1) [30], regulates ferroptosis negatively. Interestingly, its function is independent of cellular GSH levels and GPX4 activity. FSP1 catalyzes CoQ10 regeneration with NAD(P)H and influences ferroptosis progression by an independent pathway FSP1-CoQ10-NAD(P)H [31].

## Effect of ncRNA-mediated ferroptosis on tumor progression

ncRNAs are a unique class of RNAs transcribed from genes that do not encode proteins [32]. In addition to playing significant functions at the transcriptional and post-transcriptional levels, they can also govern the course of human disease through epigenetic alterations. The involvement of ncRNAs in regulating the progression of various cancer types has been well documented, and targeting ncRNAs has shown promising clinical therapeutic effects, which we will not repeat here. Recent studies have revealed that ncRNAs play an important role in regulating the progression of various cancer types through the iron death pathway, which can regulate iron death-related gene expression through epigenetic, transcriptional and translational modalities. They play a role in tumorigenesis, progression, treatment and prognosis. Although the role of ncRNAs in iron death is not yet fully defined, it has an invaluable role in the targeting of cancer therapy [33, 34]. The main relevant ncRNAs identified so far are microRNA (miRNA), long ncRNA (lncRNA) and circular RNA (circRNA).

### miRNAs and ferroptosis

miRNAs exhibit function primarily by binding to and regulating the expression of the 3′-untranslated region of the target mRNA [35]. Since more than 60% of coding genes are potential targets of miRNAs [5], miRNAs among ncRNAs are the most widely studied. miRNAs can regulate ferroptosis key molecules in various cancer cells and participate in tumor progression in numerous ways, which we have sorted it out in detail (Table 1).

**Table 1:** miRNAs regulate ferroptosis in cancer progression

miRNA	Role in ferroptosis	Mechanism	Cancer	References
miR-670-3p	Inhibit	Downregulates ACSL4	GBM	[107]
miR-23a-3p	Inhibit	Downregulates ACSL4	HCC	[108]
miR-424-5p	Inhibit	Downregulates ACSL4	OC	[40]
miR-7-5p	Inhibit	Downregulates mitoferrin	OC, OSCC and HCC	[41]
miR-7-5p	Inhibit	Upregulates ferritin, downregulates ALOX12	Cervical cancer, OSCC	[41]
exo-miR-522	Inhibit	Downregulates ALOX15	GC	[44]
miR-18a	Inhibit	Downregulates ALOXE3	GBM	[109]
miR-214-3p	Promote	Downregulates ATF4	HCC	[110]
miR-3200-5p	Promote	Downregulates ATF4	HCC	[111]
miR-155	Promote	Downregulates Foxo3a	Pancreatic cancer	[112]
miR-4735-3p	Promote	Downregulates FPN	CCRCC	[113]
exo-miR-4443	Inhibit	Downregulates m6A, Upregulates FSP1	NSCLC	[45]
miR-1228	Inhibit	Upregulates FSP1	Breast cancer	[114]
miR-4715-3p	Promote	Downregulates AURKA and GPX4	UGC	[115]
miR-9	Inhibit	Downregulates GOT1	Melanoma	[116]
miR-15a-3p	Promote	Downregulates GPX4	CRC	[117]
miR-539	Promote	Downregulates GPX4	CRC	[118]
miR-324-3p	Promote	Downregulates GPX4	Breast cancer	[37]
miR-324-3p	Promote	Downregulates GPX4	NSCLC	[119]
miR-15a	Promote	Downregulates GPX4	PCa	[120]
miR-1287-5p	Promote	Downregulates GPX4	Osteosarcoma	[121]
miR-29b	Promote	Downregulates GPX7	glioma	[122]
miR-19a	Inhibit	Downregulates IREB2	CRC	[123]
miR-130b-3p	Inhibit	Downregulates DKK1, upregulates NRF2 and HO-1	Melanoma	[124]
miR-7	Inhibit	Downregulates Keap1, upregulates NRF2	Human neuroblastoma	[125]
miR-200a	Inhibit	Upregulates NRF2	Breast cancer	[38]
miR-200a	Inhibit	Upregulates Keap1 and NRF2	ESCC	[39]
miR-6077	Promote	Downregulates NRF2	LUAD	[126]
miR-450b-5p	Promote	Downregulates NRF2	NPC	[127]
miR-365a-3p	Promote	Downregulates NRF2	NSCLC	[128]
miR-137	Inhibit	Downregulates SLC1A5	Melanoma	[129]
miR-382-5p	Promote	Downregulates SLC7A11	Ovarian, breast cancer	[130]
miR-489-5p	Promote	Downregulates SLC7A11	GC	[131]
miR-125b-5p	Promote	Downregulates SLC7A11	OSCC	[132]
miR-34c-3p	Promote	Downregulates SLC7A11	OSCC	[133]
miR-1261	Promote	Downregulates SLC7A11	HCC	[134]
miR-25-3p	Promote	Downregulates SLC7A11	PCa	[134]
miR-27a	Promote	Downregulates SLC7A11	Bladder cancer	[135]
miR-375	Promote	Downregulates SLC7A11	GC	[136]
miR-5096	Promote	Downregulates SLC7A11	Breast cancer	[137]
miR-489-3p	Promote	Downregulates SLC7A11	GC	[131]
miR-139-5p	Promote	Downregulates SLC7A12	Pancreatic carcinoma	[138]
miR-27a-3p	Inhibit	Downregulates SLC7A11	NSCLC	[139]
miR-125b-5p	Promote	Downregulates STAT3	GC	[140]
miR-101-3p	Promote	Downregulates TBLR1	LC	[141]
miR-545	Inhibit	Downregulates TF	CRC	[142]
miR-21-3p	Promote	Downregulates TXNRD1	Melanoma	[143]

Previous studies have shown that a single miRNA can be involved in ferroptosis by regulating iron death-related genes in multiple cancers simultaneously, such as miR-324-3p, miR-200a and miR-7-5p. miR-324-3p was reported to be significantly downregulated in cis-diamminedichloroplatinum II (DDP, aka cisplatin)-resistant lung adenocarcinoma cells and increased the resistant cells' sensitivity to cisplatin by targeting GPX4 [36]. Meanwhile, metformin could promote ferroptosis by the miR-324-3p/GPX4 axis in breast cancer [37]. Additionally, the miR-200 family is known for its down-regulation in human tumor cells. By targeting important mRNAs involved in epithelial mesenchymal transition (EMT) (ZEB1 and ZEB2), -catenin/Wnt signaling (-catenin), EGFR inhibitor resistance (ERRFI-1), and chemoresistance to therapeutic drugs, it plays a critical role in reducing EMT, tumor cell adhesion, migration, invasion, and metastasis. As a ferroptosis regulator, NRF2 has antioxidant properties, and its levels are regulated by Keap1. It has been reported that miR-200a regulates the Keap1/Nrf2 pathway in the mammary epithelium [38], and methylseleninic acid (MSA) can act as a chemopreventive agent for oesophageal squamous cell carcinoma (ESCC) cells by the KLF4/miR-200a/Keap1/Nrf2 axis [39]. Although miR-200a can regulate essential ferroptosis components, its involvement in ferroptosis has not been experimentally confirmed. Moreover, miR-7-5p was highly expressed in radiation-resistant ovarian, oral squamous cell carcinoma, and hepatocellular carcinoma cell lines and affected ferroptosis by downregulating the mitochondrial iron transporter protein Mitoferrin and decreasing Fe^2+^ [40]; and later, Kazuo et al. demonstrated that miR-7-5p was upregulated in radiation-resistant cells of cervical cancer and was involved in the cellular regulation of ROS, mitochondrial membrane potential, and Fe^2+^ level regulation and affects the ALOX12 and HIF1α expression [41].

miRNA is an important exosome component, and it has been detected in exosomes of several cell types [42]. 15-lipoxygenase (ALOX15) is closely associated with the accumulation of lipid ROS in cancer cells [43]. Cisplatin and paclitaxel promote miR-522 secretion by cancer-associated fibroblasts (CAFs) through the USP522/hnRNPA7 axis, thereby downregulating ALOX15 and reducing ROS production in cancer cells, ultimately leading to chemoresistance [44]. This study confirms the occurrence of ferroptosis in tumor microenvironment-associated exosomes for the first time. Moreover, exosomal miR-4443 was highly expressed in cisplatin-resistant non-small cell lung cancer (NSCLC) cells. Further studies revealed that miR-4443 could target methyltransferase-like 3 (METTL3), thereby reducing the N6 methyladenosine (m6A) level in cells, while the FSP1 expression is regulated by m6A modifications. Overall, miR-4443 regulates the FSP1 expression by METTL3 in an m6A-like manner, which in turn is involved in ferroptosis and confers cisplatin resistance to NSCLC cells [45].

To summarize Table 1 we found that different miRNAs can regulate iron ion levels through different pathways, and an imbalance of iron ions can lead to uncontrolled miRNA expression. Also, miRNAs and NRF2 exist to regulate each other. In conclusion, miRNAs are involved in potential regulatory mechanisms of ferroptosis, including various pathways such as mitochondria-associated proteins, iron metabolism, glutathione metabolism and lipid peroxidation, and in turn, miRNAs and ROS can regulate each other in various pathways.

### lncRNA and ferroptosis

lncRNA has a longer sequence than miRNA. It mainly acts as a regulator of transcription factors in the nucleus or as a sponge for miRNAs in the cytoplasm [46].

Unlike miRNAs, lincRNAs can operate as miRNA sponges to indirectly regulate the cell death process and act directly on ferroptosis key genes and proteins. The most recent research on the role of lincRNAs in ferroptosis is described in Table 2.

**Table 2:** lncRNAs regulate ferroptosis in cancer progression

IncRNA	Role in ferroptosis	Mechanism	Cancer	References
NEAT1	Inhibit	Upregulates ACSL4	NSCLC	[144]
lncRNA ASMTL-AS1	Promote	Upregulates SAT1	LUAD	[145]
NEAT1	Promote	Sponges miR-362-3p to upregulate MIOX	HCC	[146]
NEAT1	Inhibit	Downregulate SLC7A11	Melanoma	[103]
LINC00551	Promote	Sponges miR-4328 to upregulate DDIT4	LUAD	[102]
H19	Inhibit	Inhibits production of lipid ROS and induces production of GSH	Breast cancer	[147]
H19	Inhibit	Sponges miR 19b-3p to upregulate FTH1	LC	[148]
TUG1	Promote	Downregulates FTH1	Glioma	[149]
Lnc GABPB1-AS1	Promote	Downregulates GABPB1 and PRDX5	HCC	[150]
lncRNA BBOX1-AS1	Inhibit	Sponges miR-513a-3p to downregulate SLC7A11	Esophageal squamous cell cancer	[151]
LINC00618	Promote	Interacts with LSH to downregulate SLC7A11	Leukemia	[152]
P53RRA (LINC00472)	Promote	Interacts with G3BP1 to downregulate SLC7A11	LC	[99]
OIP5-AS1	Inhibit	Sponges miR-128-3p to upregulate SLC7A11	Prostate cancer	[153]
lncRNA slc16a1-AS1	Inhibit	Sponges miR-143-3p to upregulate SLC7A11	Renal cell carcinoma	[154]
HEPVAL	Promote	Downregulate SLC7A11	HCC	[155]
lncFERO	Inhibit	Interacts with hnRNPA1 to upregulate SCD1	GC	[47]
lncBDNF-AS	Inhibit	Interacts with WDR5 and FBXW7 to upregulate VDAC3	GC	[156]
RP11-89	Inhibit	Sponges miR-129-5p to upregulate PROM2	Bladder cancer	[157]
lncLASTR	Inhibit	Upregulates GPX4	Stomach adenocarcinoma	[158]
lncPVT1	Inhibit	Sponges miR-214-3p to upregulate GPX4	HCC	[52]
HCG18	Inhibit	Sponges miR-450b-5p to upregulate GPX4	HCC	[159]
MEG8	Inhibit	Sponges miR-497-5p to upregulate NOTCH2	Benign hemangioma	[160]
lncRNA TMEM161B-AS1	Inhibit	Sponges mir-27a-3p to upregulate FANCD2 and CD44	Glioma	[161]
lncRNA MT1DP	Promote	Sponges miR-365a-3p to downregulate NRF2	NSCLC	[128]
LINC01606	Inhibit	Sponges miR-423-5p to upregulate SCD1	Colon cancer	[162]
LINC00336	Inhibit	Sponges miR6852 to upregulate CBS	LC	[163]
LINC01564	Inhibit	Upregulate NFE2L2	Glioma	[164]

Stearoyl coenzyme A desaturase 1 (SCD1) is a mechano reactive enzyme that reprograms lipid metabolism in gastric cancer stem cells (GCSC) and participates in ferroptosis. In contrast, exosomal lncFERO (exo-lncFERO) regulates SCD1 mRNA levels, causing PUFA dysregulation and subsequent ferroptosis inhibition. This enhances dryness and regulates chemosensitivity in the body [47].

lncPVT1 is upregulated in various cancers [48–50]. It is involved in tumor cell proliferation, migration, autophagy, apoptosis, and EMT. It promotes the malignant progression of tumors through physiological or pathological mechanisms like hypoxia and exosomes [50, 51], which are potential therapeutic targets for human cancers. According to studies, the therapeutic anesthetic ketamine can limit hepatocarcinoma viability and induce ferroptosis. Moreover, lncPVT1 can interact with miR-214-3p and hinder it from acting as a sponge for GPX4, effectively responding to ketamine-induced ferroptosis [52].

Cancer genomic databases and bioinformatics analysis have identified many differentially expressed IncRNAs with prognostic value associated with ferroptosis [53–55]. However, these IncRNAs still lack experimental confirmation of their potential as ferroptosis markers.

Overall, lncRNAs can affect ROS metabolism directly or indirectly through a variety of mechanisms including GPX4, ferric ions, SLC7A11 and, conversely, lncRNAs are regulated by them.

### circRNA and ferroptosis

CircRNA is a single-stranded RNA molecule in a covalently closed loop. Therefore, it is nucleic acid exonuclease resistant and exhibits high stability in the body [56]. Simultaneously, its high abundance is tissue- and stage-specific [57]. This provides an advantage for circRNAs to act as biomarkers and targets for cancer therapy.

Several studies have revealed a relationship between circRNA and ferroptosis. circRNAs can mediate ferroptosis through multiple mechanisms in many tumor types (Table 3). Compared to the nucleus, circRNAs are more often found in the cytoplasm and act as sponges for miRNAs that regulate the target genes' expression [58].

**Table 3:** circRNAs regulate ferroptosis in cancer progression

circRNA	Role in ferroptosis	Mechanism	Cancer	References
Hsa_circ_0021087 (circLMO1)	Promote	Sponges miR-4291 to upregulate ACSL4	Cervical cancer	[165]
circGFRA1	Inhibit	Sponges miR‐1228 to upregulate AIFM2	Breast cancer	[114]
Circ clARS	Promote	Interacts with ALKBH5	HCC	[101]
CircABCB10	Inhibit	Sponges miR-326 to upregulate CCL5	Rectal cancer	[166]
Circ_0008035	Inhibit	Sponges miR-599 to upregulate EIF4A1	GC	[167]
circPVT1	Inhibit	Sponges miR-30a-5p to upregulate FZD3	Esophageal cancer	[168]
circ_0007142	Inhibit	Sponges miR-874-3p, upregulates GDPD5	CRC	[169]
circKIF4A	Inhibit	Sponges miR-1231 to upregulate GPX4	TPC	[66]
circDTL	Inhibit	Sponges miR-1287-5p to upregulate GPX4	NSCLC	[100]
CircIL4R	Inhibit	Sponges miR-541-3p to upregulate GPX4	HCC	[119]
Circ-TTBK2	Inhibit	Sponges miR-761 to upregulate ITGB8	Glioma	[170]
Circ_0000745	Inhibit	Sponges miR-494-3p to upregulate NET1	ALL	[62]
circCDK14	Inhibit	Sponges miR-3938 to upregulate PDGFRA	Glioma	[171]
circKDM4C	Promote	Sponges miRNA let-7b-5p to upregulate p53	AML	[172]
circ0097009	Inhibit	Sponges miR-1261 to upregulate SLC7A11	HCC	[134]
circEPSTI1	Inhibit	Sponges miR-375, miR-409-3p and miR-515-5p to upregulate SLC7A11	Cervical cancer	[173]
circFNDC3B	Inhibit	Sponges miR-520d-5p to upregulate SLC7A11	OSCC	[174]
circ_0067934	Inhibit	Sponges miR-545-3p to upregulate SLC7A11	Papillary and follicular thyroid cancers	[175]
circ-BGN	Inhibit	Upregulates OTUB1 and SLC7A11	Breast cancer	[59]
circFOXP3	Inhibit	Sponges miR-7a-11p to upregulate SLC520A5	LC	[176]
circRHOT1	Inhibit	Sponges miR-106a-5p to upregulate STAT3	Breast cancer	[177]
circ_0000190	Promote	Sponges miR-382-5p to upregulate ZNRF3	GC	[178]

Tumor resistance can significantly compromise clinical efficacy. circ-BGN was first found to be highly expressed in trastuzumab-resistant HER2-positive breast cancer. Further studies revealed that circ-BGN could act directly on SLC7A11, a core molecule of ferroptosis, and enhanced OTUB1-mediated deubiquitination of SLC7A11, thereby inhibiting ferroptosis. The conclusion was also confirmed by in vivo experiments [59]. hsa_circ_0000745 has the potential to act as a diagnostic marker for cervical cancer, gastric cancer, and other cancers [60, 61]. Yanbi et al. recently found that circ_0000745 involves cell cycle progression, glycolytic metabolism, apoptosis, and ferroptosis in acute lymphoblastic leukemia. Furthermore, this role is accomplished through the circ_0000745/miR-494-3p/NET1 axis [62]. It has been reported that circKIF4A can promote numerous tumor progressions and mediate glycolytic metabolism and drug resistance through competitive endogenous RNA mechanism mechanism [63–65]. In papillary thyroid cancer, circKIF4A negatively regulates ferroptosis and promotes tumor proliferation in vitro and in vivo. In essence, circKIF4A can absorb miR-1231 to increase GPX4 levels [66].

In general, circRNAs could be potential therapeutic targets for the treatment of cancer through the ferroptosis pathway.

In this section, we systematically summarize the ncRNAs associated with ferroptosis in cancer to date and explore the regulatory role of ncRNAs in cancer progression and iron death, which implies that ncRNAs have great potential as anti-cancer therapeutic targets through regulation of ferroptosis. Moreover, ferroptosis-related ncRNAs are individually heterogeneous across tumors, which has significant implications for personalised tumor therapy.

Despite the full potential of ferroptosis-related ncRNAs, there are still many unanswered questions. Although a clear regulatory role for ncRNAs in the development of ferroptosis in tumors has been identified, little is still known about the in-depth mechanisms underlying this component. This makes the clinical application of ncRNA-dependent approaches to ferroptosis a major obstacle. Furthermore, to translate basic research into clinical trials, the construction of additional animal models to validate the role of ncRNAs in ferroptosis is a must. In addition, given the shortcomings of conventional treatment options for tumors, research on the application of biomaterials such as molecular nanomaterials for targeted tumor ferroptosis therapy is urgently needed. Besides, due to the diversity of ncRNA biological functions, targeting ncRNA therapy is likely to cause some complications and cause damage to non-tumor organs. For example, miR-375-3p and miR-214-3p, which have the potential to both promote ferroptosis in tumor cells of cervical cancer and HCC, may also cause fibrosis of cardiomyocytes and acute renal impairment[67, 68]. It is therefore important to achieve tumor-targeted metastasis of ncRNAs, and multidisciplinary cross-fertilisation will facilitate this process.

## Relationship between ferroptosis and other PCDs

Abnormal cell death regulation is an important feature of cancer. PCDs are highly involved in tumor development, including apoptosis, necroptosis, autophagy, pyroptosis, ferroptosis, and cuproptosis. Therefore, exploring the mechanisms of different types of cell death is of great importance in cancer. Researchers have discovered that ferroptosis is independent and connected to other types of cell death and that its essential regulators are also involved in regulating other types of cell death [69]. These death types usually share a common pathway [70]. Consequently, further investigation of the inter regulation of ferroptosis with other types of programmed cell death and developing strategies that can trigger numerous planned cell deaths are extremely promising cancer treatment strategies.

### Apoptosis and ferroptosis

Apoptosis is a form of cellular suicide induced by the activation of intracellular death programs and was initially thought to be the only way of PCD. It is an intrinsic tumor suppressor mechanism that physically displays cellular crumpling, chromatin aggregation, and the production of apoptotic vesicles followed by phagocytosis [2]. Mechanistically, apoptosis consists of three main aspects: oxidative damage, imbalance of calcium homeostasis and mitochondrial damage. Apoptosis can be initiated by ncRNAs through regulation of the relevant receptors or as cerRNAs.

Death structural domain-associated protein (Daxx) mediates apoptosis through the Fas-Daxx-ASK1-JNK1 axis, while the ferritin FTH1 inhibits the action of Daxx [71]. Ferroptosis inducer erastin activates the C/EBP homogenic protein (CHOP) signal pathway, affecting the expression of p53 non-dependent PUMA and increasing sensitivity to tumor necrosis factor-related apoptosis-inducing ligand (TRAIL) induced cell death [72]. Furthermore, apoptosis may be directly transformed into ferroptosis [73].

### Necroptosis and ferroptosis

Necroptosis is an alternate cell death mechanism triggered when apoptosis is blocked and is a degenerative pathology caused by damaging factors. Morphological features include cell swelling, membrane rupture, release of cytoplasmic contents and chromosome condensation. The basic molecular mechanism consists of receptor-interacting kinases (RIPK1 and RIPK3) and mixed-spectrum kinase structural domain-like pseudokinases (MLKL). The RIPK1/RIPK3 complex recruits and phosphorylates MLKL translocates to the plasma membrane, and forms channels, releasing damage-associated molecular patterns (DAMPs), permeabilization of the plasma membrane, and release of contents [74].

By activating the mitochondrial permeability transition pore (MPTP) and phosphorylating RIPK1, iron excess induces necrotic apoptosis in ischemic stroke. Heat shock protein 90 (HSP90) is an evolutionarily conserved and commonly expressed molecular chaperone. It intensifies RIPK1 phosphorylation, inhibits GPX4 activity, and can induce necroptosis and ferroptosis [75]. Thus, HSP90 acts as a co-regulatory node for necroptosis and iron sagging. ferroptosis and necroptosis are known to be positively regulated by ACSL4 and MLKL, respectively. In a mouse model of renal ischemia–reperfusion injury, ACSL4 and MLKL knockdown modulate the sensitivity of necroptosis and ferroptosis, respectively [76]. This led us to wonder if ferroptosis and necroptosis have complementing processes reasonably. Therefore, it is essential to continue to explore the relationship between ferroptosis and necroptosis.

### Autophagy and ferroptosis

Autophagy is a process by which cells ‘self-feed’. Under physiological conditions, basal autophagy is a cellular self-protection mechanism, while induced autophagy under stressful conditions may cause cell death. Morphologically, it is characterised by the accumulation of autophagic vesicles and cytoplasmic vesiculation without chromatin condensation [77]. There are three main forms of autophagy: microautophagy, macroautophagy, and chaperone-mediated autophagy (CMA). Autophagy begins mechanistically with pre-autophagic structures in the cytoplasm, which create autophagosomes after phagocytosis of damaged organelles and denatured macromolecules. Subsequently, autophagosomes combine with lysosomes to generate autolysosomes, which destroy the contents of autophagosomes [77].

In exploring the relationship between autophagy and ferroptosis, we once again identified HSP90. HSP90 increases the protein stability of CMA receptor lysosome-associated membrane protein 2A (LAMP2A) to accelerate GPX4 degradation and enhance ferroptosis [78]. Zili et al. found that increased BECN1 mRNA stability with the involvement of ELAVL1 caused ferritin phagocytosis and subsequent ferroptosis [79]. While in Parkinson's disease (PD), FTH1 overexpression inhibits ferritin phagocytosis and, ultimately, ferroptosis [80]. We, therefore, hypothesize that ferritin phagocytosis (a sort of selective autophagy) may have a good connection with ferroptosis. Nuclear receptor coactivator 4 (NCOA4) has been reported to be involved in autophagy-dependent ferritin degradation [81], and NCOA4 overexpression can contribute to ferritin degradation and promote increased free iron and subsequent ferroptosis [82]. Interestingly, intracellular free iron regulates NCOA4 levels [81]. Moreover, RAB7A and SQSTM1 are regulators of lipophagy and clockophagy, respectively, and their downregulation prevents lipid peroxidation-dependent ferroptosis [83, 84]. High mobility group box-1 protein (HMGB1) is a DAMP, and its relationship with autophagy and ferroptosis is more complex. On one side, autophagy-dependent ferroptosis can increase the HMGB1 release [85], whereas HMGB1 can be engaged in the advancement of autophagy and ferroptosis [86, 87]. Recent studies have revealed that hippocampal calmodulin-like 1 (HPCAL1) is an autophagy receptor that affects membrane tension by regulating CDH2, which further affects lipid peroxidation and ultimately inhibits ferroptosis in vitro and in vivo [88]. Another autophagy receptor, Tax1 (human T cell leukemia virus type I) binding protein 1 (TAX1BP1), promotes GPX4 degradation and subsequent ferroptosis in response to copper stress [89]. The above studies suggest a close association between autophagy and ferroptosis.

### Pyroptosis and ferroptosis

Programmed cell death induced by inflammatory vesicles mediated by gasdermins is known as cell scorch death and can amplify local or systemic inflammatory effects [90]. Unique to cell death by scorch is the formation of many bubble-like protrusions, known as scorch vesicles, within the cell. Mechanistically, inflammatory vesicles sense danger and recruit and activate caspase 1, which stimulates inflammatory proteins that cleave gastrin D (GSDMD), causing it to attach to the cell membrane and generate pores, which is the conventional mechanism of scorch death. The non-classical pathway of scorch death is mainly mediated by cystatase-4, caspase-5, and caspase-11 [91].

We found that there are multiple co-stimulatory factors for scorch death and ferroptosis. Transcription factor P53 is an important regulatory molecule of ferroptosis. Moreover, in NSCLC, P53 can directly increase scorch death and inhibit tumor growth [92]. In a myocardial fibrosis model, MLK3 regulates ferroptosis and scorch death through the JNK/p53 pathway and the NF-κB/NLRP3 pathway, while miR-351 can inhibit MLK3 expression [93]. Additionally, elevated ferric ions and ROS levels can induce scorch death and ferroptosis. Rui et al. found synergistic effects of scorch death and ferroptosis using dual-induced nano drugs [94]. Furthermore, iron-activated ROS can induce scorch death in melanoma through the Tom20-Bax-caspase-GSDME axis [95]. Another study found that in macrophages, GPX4, a core regulatory protein of ferroptosis, can block GSDMD activity and trigger scorch death by reducing lipid peroxidation. Interestingly, HMGB1 levels were thus altered, eventually leading to sepsis [96]. In conclusion, the regulatory relationship between scorch death and ferroptosis should be explored in depth.

### Cuproptosis and ferroptosis

Copper is a key factor in cell signaling, and cell death induced by copper overload was found to be a new form of cell death called cuproptosis. The main targets of copper death are the mitochondria, which are morphologically characterised by mitochondrial wrinkling and mitochondrial membrane rupture. Both copper ion carrier induction and dysregulation of copper homeostasis lead to copper death. Copper binds to lipases in the tricarboxylic acid (TCA) cycle, leading to protein aggregation, proteotoxic stress, and cell death [97].

Elesclomol (ES) is a copper ion carrier. In CRC cells, ES allows copper ions to be retained in mitochondria, leading to ROS accumulation, promoting SLC7A11 degradation, and increasing susceptibility to ferroptosis [98]. Given the novelty of cuproptosis, its relationship with ferroptosis has not been extensively studied.

**Fig. 2:**
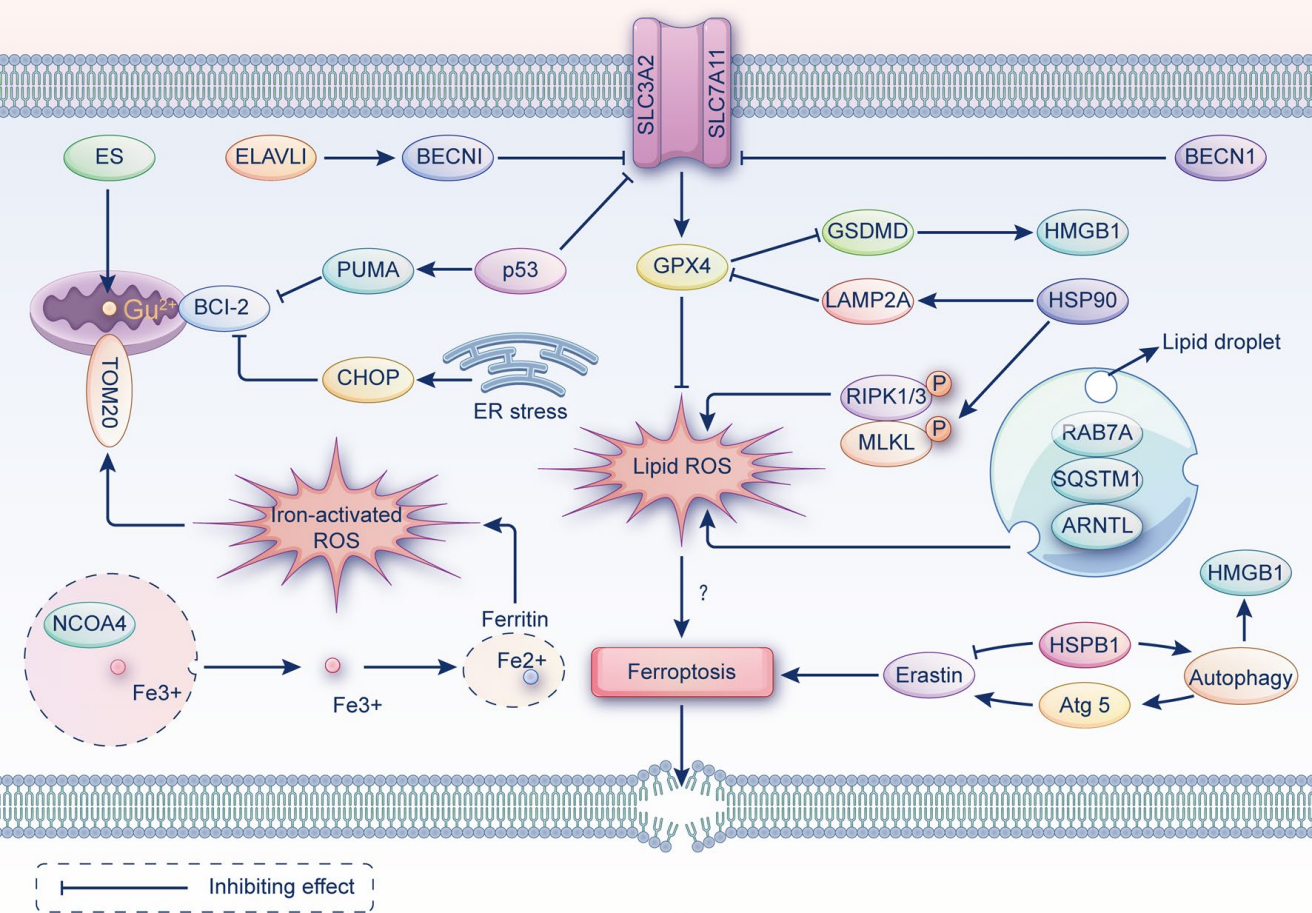
The mutual regulatory mechanisms between ferroptosis and other forms of death. The various initiators and effector molecules involved in ferroptosis, apoptosis, necroptosis, autophagy, pyroptosis and cuproptosis can interact to promote cell death

Based on the initial investigation, we have generated Fig. 2, in which molecules such as HSP90, HMGB1, and P53 show multiple times. Thus, are there shared regulatory proteins and signaling pathways between ferroptosis and other PCDs? Is this sharing related to the positive correlation between ferroptosis and other forms of death? Can we suppress multiple death pathways through this sharing? Hopefully, these questions can be addressed in subsequent studies. Although many of the study subjects are non-tumor disorders, this suggests the complexity of the relationships between ferroptosis and other PCDs, hence pointing the way for future tumor-related research.

## Role of ncRNA in crosstalk between ferroptosis and other PCDs in tumors

ncRNAs are important regulators of eukaryotic gene expression, and many ncRNAs have been found to mediate PCD to influence tumor malignant progression. The data above demonstrate the relationship and similarities between ferroptosis and numerous forms of cell death. Without a doubt, ncRNAs participate in regulating crosstalk between these PCDs. This section provides a summary of relevant studies **(**Table 4**)**.

**Table 4:** Role of ncRNAs in crosstalk between ferroptosis and other models of cell death in tumors

ncRNA	Role in PCDs	Mechanism	Cancer	References
lncRNA NEAT1	Promote ferroptosis and apoptosis	Sponges miR-362-3p to upregulate MIOX	HCC	[146]
lncRNA P53RRA (LINC00472)	Promote ferroptosis and apoptosis	Interacts with G3BP1 to downregulate SLC7A11	LC	[99]
lncRNA OIP5-AS1	Inhibit ferroptosis and apoptosis	Sponges miR-128-3p to upregulate SLC7A11	PCa	[153]
lncRNA HCG18	Inhibit ferroptosis and apoptosis	Sponges miR-450b-5p to upregulate GPX4	HCC	[159]
lncRNA TMEM161B-AS1	Inhibit ferroptosis and apoptosis	Sponges mir-27a-3p to upregulate FANCD2 and CD44	Glioma	[161]
LINC01564	Inhibit ferroptosis and apoptosis	Upregulate NFE2L2	Glioma	[164]
CircABCB10	Inhibit ferroptosis and apoptosis	Sponges miR-326 to upregulate CCL5	Rectal cancer	[166]
circDTL	Inhibit ferroptosis and apoptosis	Sponges miR-1287-5p to upregulate GPX4	NSCLC	[100]
Circ_0000745	Inhibit ferroptosis and apoptosis	Sponges miR-494-3p to upregulate NET1	ALL	[62]
circRHOT1	Inhibit ferroptosis and apoptosis	Sponges miR-106a-5p to upregulate STAT3	Breast cancer	[177]
circ_0007142	Inhibit ferroptosis and apoptosis	Sponges miR-874-3p, upregulates GDPD5	CRC	[169]
Hsa_circ_0021087 (circLMO1)	Promote ferroptosis and apoptosis	Sponges miR-4291 to upregulate ACSL4	Cervical Cancer	[165]
LINC00618	Promote ferroptosis in a manner dependent upon apoptosis	Interacts with LSH to downregulate SLC7A11	leukemia	[152]
NEAT1	Inhibit ferroptosis and autophagy	upregulate SLC7A11	melanoma	[103]
LINC00551	Promote ferroptosis in a manner dependent upon autophagy	Sponges miR-4328 to upregulate DDIT4	LUAD	[102]
lncRNA H19	Inhibit autophagy-mediated ferroptosis	Inhibits production of lipid ROS and induces production of GSH	Breast cancer	[147]
Circ clARS	Promote autophagy-mediated ferroptosis	Interacts with ALKBH5	HCC	[101]

Zuli et al. found that LINC00618 promotes apoptosis by increasing BCL2-related X (BAX) levels and cleaved caspase-3 and by repressing SLC7A11 transcription through lymphatic-specific decapping enzymes (LSH) to promote ferroptosis. However, ferroptosis initiated by LINC00618 depends on vincristine (VCR)-triggered apoptosis. Thus, LINC00618 promotes ferroptosis in an apoptosis-dependent manner [99]. Additionally, many ncRNAs are involved in cancer progression by simultaneously regulating apoptosis and ferroptosis. For example, the methylation-modified lncRNA P53RRA is down-regulated in lung cancer and promotes nucleoplasmic translocation of p53 by interacting with G3BP1, ultimately leading to cell cycle arrest, apoptosis, and ferroptosis [99]. Another study found that the oncogenic factor circDTL upregulates GPX4 by acting as a ceRNA competing for binding with miR-1287-5p, ultimately inhibiting ferroptosis and apoptosis [100].

The link between ferroptosis and autophagy appears to be closer. ALKBH5 is a negative regulator of autophagic flux, and cIARS decreases ferroptosis via inhibiting ALKBH5-mediated autophagy, which increases sorafenib (SF) resistance in HCC cells [101]. Oncology studies have shown that LINC00551 inhibits cell viability in lung adenocarcinoma (LUAD). Mechanistically, LINC00551 inhibits mTOR activity through the miR-4/DDIT4 signaling pathway, upregulates autophagy levels, and then promotes ferroptosis in an autophagy-dependent manner [102]. Recent studies have found that lincRNA NEAT1 is involved in ferroptosis and autophagy induced by gambogenic acid (GNA), a natural anticancer compound, through SLC7A11 / GPX4 and AMPK / mTOR axis in melanoma [103].

With the preceding data, we hypothesize that ferroptosis, apoptosis, and autophagy have synergistic effects. However, there are few reports on the ncRNAs regulation in tumors in the crosstalk between ferroptosis and other PCDs, and the corresponding regulatory relationships still need further study.

## Conclusion

Recently, there has been considerable interest in developing cancer drugs targeting the PCD pathway. Besides, ferroptosis has attracted much attention as a newly discovered form of cell death. Although ferroptosis research has surged in recent years, many questions remain unresolved. To address the direction of this review, the following questions and perspectives are presented.

First and foremost, the ultimate triggering cause for ferroptosis is unknown. Although iron and lipid peroxide accumulation are critical stages, not all lipid peroxidation damage leads to cellular ferroptosis. Then, it remains to be investigated whether lipid peroxidation reaches a certain threshold to cause plasma membrane rupture directly; or needs to be activated by some unknown molecule to cause the final effect phase.

Although a growing number of ncRNAs have been linked to the regulation of ferroptosis, the regulatory mechanisms remain poorly understood. Furthermore, there is still a lack of ferroptosis-specific markers for clinical diagnosis. Notably, novel small ncRNAs such as PIWI-interacting RNA (piRNA) and tRNA-derived small RNA (tsRNA) have been shown to have biological functions in cancer. What role do they play in ferroptosis?

Endoplasmic reticulum (ER) stress, redox stress, and mitochondrial dysfunction appear to be common pathways for multiple death types [104]. Investigating the biological relevance of ferroptosis to other PCDs is of great interest. Nevertheless, the findings discussed in Part V indicate the complexity of this relationship. Furthermore, there are limited investigations on the role of ncRNAs in the crosstalk between ferroptosis and other forms of crosstalk. Future research may reveal if we may adversely regulate many death pathways through a single target.

The advantages of ncRNA as tumour prevention, monitoring treatment response and prognosis have been illustrated in the literature and have yielded some promising results in the clinic [105]. However, the clinical application of ferroptosis and thus tumour suppression through an ncRNA-dependent approach faces significant obstacles. On the one hand, the lack of understanding of specific mechanisms has led to limited application of ncRNA modifying agents in ferroptosis. On the other hand, although promoting cellular ferroptosis can inhibit tumour progression, will it be accompanied by damage to other non-tumour organs or fibrosis? In addition, ncRNA-based therapies inherently have many limitations, such as instability and tolerability [106]. Due to the instability of ncRNAs, the mode of transport has a significant impact on the efficiency of transport. Currently, nanoparticle-based, phage-based and other delivery methods are being optimized. Also, ncRNAs, being RNAs, are likely to be recognized and cleared by the immune system. It is hoped that the next generation of ncRNA therapies will overcome these drawbacks and allow for real clinical applications.

## Data Availability

Not applicable.

## Abbreviations

ncRNAs: Non-coding RNAs
PCD: Programmed cell death
GSH: Glutathione
Tf: Transferrin
LTF: Lactotransferrin
TfR: Transferrin receptor
STEAP3: Six transmembrane epithelial antigen of protein 3
SLC39A14/ZIP14: Solute carrier family 39 member 14
SLC39A8/ZIP8: Solute carrier family 39 member 8
NTBI: Non-transferrin-bound iron
PCBP1/2: Poly C-binding protein 1/2
FTL: Light chain
FTH1: Heavy chain 1
IRP1/2: Iron regulatory protein
HO-1: Heme oxygenase 1
Nrf2 / NFE2L2: Transcription factor nuclear factor E2-related factor 2
SLC40A1 / ferroportin1 / FPN: Iron efflux protein solute carrier family 40 member 1
ROS: Reactive oxygen species
PUFAs: Polyunsaturated fatty acids
AA: Arachidonic acid
AdA: Adrenoic acid
ACSL4: Acyl coenzyme A synthase long chain family member 4
LPCAT3: Lysophosphatidylcholine acyltransferase 3
AA / AdA-PE: AA/AdA-phosphatidylethanolamine
AA / AdA-OOH-PE: AA/AdA-hydroperoxide-PE
MDA: Malondialdehyde
4-HNEs: 4-Hydroxynonenal
COX2: Cyclooxygenase-2
NOX2: Nicotinamide adenine dinucleotide phosphate oxidases 2
SLC7A11: Solute carrier family 7 member 11
SLC3A2: Solute carrier family 3 member 2
GPX4: Glutathione peroxidase 4
L-OOH: Peroxides
L-OH: Alcohols
NADPH: Nicotinamide adenosine dinucleotide hydrogen phosphate
RSL3: RAS selective lethal small molecule 3
DPP4: Dipeptidyl peptidase 4
CDKN1A/p21: Cell cycle protein-dependent kinase inhibitor 1A
AIFM2: Apoptosis-inducing factor mitochondrial-associated 2
FSP1: Iron death inhibitory protein 1
miRNA: MicroRNA
lncRNA: Long ncRNA
circRNA: Circular RNA
DDP, aka cisplatin: Cis-diamminedichloroplatinum II
MSA: Methylseleninic acid
ESCC: Oesophageal squamous cell
ALOX15: 15-Lipoxygenase
CAFs: Cancer-associated fibroblasts
METTL3: Methyltransferase-like 3
m6A: Methyladenosine
CSCs: Cancer stem cells
SCD1: Stearoyl coenzyme A desaturase 1
GCSC: Gastric cancer stem cells
exo-lncFERO: Exosomal lncFERO
EMT: Epithelial mesenchymal transition
Daxx: Death structural domain-associated protein
CHOP C/EBP: Homogenic protein
TRAIL: Tumor necrosis factor-related apoptosis-inducing ligand
RIPK1 and RIPK3: Receptor-interacting kinases
MLKL: Mixed-spectrum kinase structural domain-like pseudokinases
DAMPs: Damage-associated molecular patterns
MPTP: Mitochondrial permeability transition pore
HSP90: Heat shock protein 90
CMA: Chaperone-mediated autophagy
LAMP2A: Lysosome-associated membrane protein 2A
PD: Parkinson’s disease
NCOA4: Nuclear receptor coactivator 4
HMGB1: High mobility group box-1 protein
HPCAL1: Hippocampal calmodulin-like 1
TAX1BP1: Tax1 (human T cell leukemia virus type I) binding protein 1
GSDMD: Gastrin d
TCA: Tricarboxylic acid
ES: Elesclomol
BAX: BCL2-related X
LSH: Lymphatic-specific decapping enzymes
VCR: Vincristine
SF: Sorafenib
LUAD: Lung adenocarcinoma
GNA: Gambogenic acid
piRNA: PIWI-interacting RNA
tsRNA: TRNA-derived small RNA
ER: Endoplasmic reticulum
COAD: Colorectal cancer
GBM: Glioblastoma
GC: Gastric cancer
OSCC: Oral squamous cell carcinomas
CRC: Colorectal cancer
OC: Ovarian cancer
CCRCC: Clear cell renal cell carcinoma
UGC: Upper gastrointestinal adenocarcinoma
TPC: Papillary thyroid cancer
PCa: Prostate cancer
NPC: Nasopharyngeal carcinoma
ALL: Acute lymphoblastic leukemia
AML: Acute myeloid leukemia
